# 血清及脑脊液肿瘤标志物在非小细胞肺癌软脑膜转移辅助诊治中的价值

**DOI:** 10.3779/j.issn.1009-3419.2020.103.09

**Published:** 2020-06-20

**Authors:** 永娟 林, 会颖 李, 明敏 黄, 震宇 尹, 剑卿 吴

**Affiliations:** 1 210029 南京，南京医科大学附属第一临床医学院 Department of Geriatric, the First Affiliated Hospital of Nanjing Medical University, Nanjing 210029, China; 2 210008 南京，南京医科大学附属鼓楼临床医学院 Department of Geriatric, Nanjing Drum Tower Hospital Clinical College of Nanjing Medical University, Nanjing 210008, China

**Keywords:** 肺肿瘤, 软脑膜转移, 脑脊液, 肿瘤标志物, 诊断, Lung neoplasms, Leptomeningeal metastasis, Cerebrospinal fluid, Tumor markers, Diagnosis

## Abstract

**背景与目的:**

软脑膜转移（leptomeningeal metastasis, LM）是指恶性肿瘤细胞浸润软脑膜，并在脑脊液（cerebrospinal fluid, CSF）中播散，预后极差，是晚期非小细胞肺癌（non-small cell lung cancer, NSCLC）患者致死的重要原因之一，因此早期的诊断和及时的治疗具有重要意义，CSF细胞学是LM诊断的金标准，但常常伴随着检测敏感性低、无法评估疗效等问题。本文旨在探讨血清及CSF中肿瘤标志物（tumor markers, TM）在NSCLC伴LM患者诊治的临床价值。

**方法:**

选取NSCLC伴LM患者19例，另选同期27例神经系统良性肿瘤（nonmalignant neurological diseases, NMNDs）患者作为对照组。观察比较两组患者血清和CSF中癌胚抗原（carbohydrate antigen, CEA）、糖类抗原125（carbohydrate antigen-125, CA125）、细胞角蛋白19片断抗原（cytokeratin 19 fragments, CYFRA21-1）和神经元烯醇化酶（neurone specific enolase, NSE）检测水平和检出阳性率，比较不同组TM的敏感性和特异性，并分析血清与CSF中TM检出情况相关性，最后动态监测2例LM患者血清和CSF中TM水平，分别评估颅外和颅内治疗疗效。

**结果:**

LM组CSF和血清中TM水平和检出阳性率均高于对照组（*P* < 0.05），同时LM组CSF中CEA、CYFRA21-1、NSE水平高于血清，差异有统计学意义（*P* < 0.05）。CSF中TM检出阳性率与血清差异不具有统计学意义（*P* > 0.05）。CSF中CYFRA21-1敏感性最高（88.2%），CEA特异性最好（92.3%），联合指标中CEA或NSE任一项超过临界值则敏感性和阴性预测值为100%，特异性为74.1%。CYFRA21-1和NSE同时超过临界值时特异性和阳性预测值为100%，敏感性为78.9%。亚组分析显示，CSF细胞学阳性人群TM检出阳性率超过有磁共振成像（magnetic resonance imaging, MRI）异常的人群，但不具有统计学差异（*P* > 0.05）。LM组血清与CSF中TM检出阳性率不一致。另外，脑室中CSF与腰穿中CSF具有相同的生化性质，动态监测血清和CSF中TM浓度，可分别评估颅外和颅内病灶的疗效。

**结论:**

血清和CSF中TM为NSCLC伴LM患者增加了一个早期辅助诊断指标，动态监测可评估治疗疗效，值得临床推广应用。

## Abstract (en)

**Background and Objective:**

Leptomeningeal metastasis (LM) is one of the most common causes of death in patients with advanced non-small cell lung cancer (NSCLC), which is defined as malignant cells spreading to meninges and cerebrospinal?uid (CSF). Therefore, early diagnosis and timely treatment are essential. CSF cytology is the gold standard for LM diagnosis, however, it has a low sensitivity for diagnosis and can't be used to evaluate the treatment effect. The aim of this study was to assess the clinical value of serum and CSF tumor markers (TM) in the diagnosis and treatment of NSCLC patients with LM.

**Methods:**

Nineteen patients with NSCLC-LM and 27 patients with nonmalignant neurological diseases (NMNDs) were included. We tested the levels and positive rates of carbohydrate antigen (CEA), carbohydrate antigen-125 (CA125), cytokeratin 19 fragments (CYFRA21-1) and neurone specific enolase (NSE) in CSF and serum, compared the sensitivity and specificity in the diagnosis of LM between different groups, and analyzed the correlation of detection between serum and CSF. Finally, we measured serum and CSF TM dynamically in 2 patients with NSCLC developing LM in an attempt to correlate these with the treatment response of extracranial and intracranial, respectively.

**Results:**

The levels and positive rates of TM in CSF and serum in LM group were higher than those in NMNDs (*P* < 0.05). In LM group, the levels of CEA, CYFRA21-1 and CEA were significantly higher in CSF than that in the serum (*P* < 0.05), whereas, there was no statistical significance in positive rates of TM between CSF and serum (*P* > 0.05). In CSF, CYFRA21-1 has the highest sensitivity (88.2%) and CEA has the best specificity (92.3%) to distinguish patients between LM and NMNDs. For combined detection of CEA, CA125, CYFRA 21-1 and NSE in CSF, when at least CEA or NSE was positive in patients with LM, the sensitivity and negative predictive value were 100.0%, and the specificity was 74.1%. When both CYFRA21-1 and NSE were positive, the specificity and positive predictive value were 100.0%, and the sensitivity was 78.9%. Furthermore, subgroup analysis showed that the detection rates of TM in CSF cytology positive population was higher than that in typical abnormalities magnetic resonance imaging population, but there was no statistical difference (*P* > 0.05). The detection of TM between serum and CSF in LM patients had no significant correlation. Moreover, biochemical properties of CSF from ventricle and lumbar puncture are similar, therefore evaluating the levels of TM in serum and CSF dynamically can be used to assess the extracranial and intracranial treatment effect, respectively.

**Conclusion:**

Our study demonstrates that Serum and CSF TM can work as an auxiliary clinical diagnostic tool, which has a potential value in early diagnosis of NSCLC patients with LM. Serial measurement of TM may play an important role in the clinical management of NSCLC patients with LM, which is worthy of further promotion and clinical application.

软脑膜转移（leptomeningeal metastasis, LM）是晚期非小细胞肺癌（non-small cell lung cancer, NSCLC）最严重的并发症之一，发生率约为5%^[1]^，随着表皮生长因子受体拮抗剂（epidermal growth factor receptor-tyrosine kinase inhibitors, EGFR-TKIs）的广泛应用，NSCLC患者生存期显著延长，LM的发生率也随之增加。据报道，LM在*EGFR*突变人群中发生率达到9.4%^[2]^。LM病情进展迅速，预后极差，中位生存期不足3个月^[3]^，早期的诊断和及时的治疗具有重要的临床意义。目前LM诊断的金标准仍然是脑脊液（cerebrospinal fluid, CSF）细胞学检查，但阳性率低，且检查结果会受标本的采集、检测时间以及检测方法的影响^[4]^。头颅磁共振成像（magnetic resonance imaging, MRI）可进一步提高LM诊断的敏感性，但神经影像学上LM病灶往往为软脑膜的增厚、结节，临床无法动态检查MRI评估疗效^[5]^。外周血肿瘤标志物（tumor markers, TM）是肺癌早期诊断和动态评估疗效的重要指标，CSF中TM同样稳定^[6]^，但是作为LM辅助诊断是否具有较高的敏感性和特异性，以及是否可以动态评估有待证实。因此，我们对临床用于肺癌诊断的4项血清TM [癌胚抗原（carbohydrate antigen, CEA）、糖类抗原125（carbohydrate antigen-125, CA125）、细胞角蛋白19片断抗原（cytokeratin 19 fragments, CYFRA21-1）和神经元烯醇化酶（neurone specific enolase, NSE）]进行CSF和血清的同步检测，目的是对此4种TM在肺癌LM辅助诊断中的应用进行评价，旨在提高肺癌LM辅助诊断的敏感性，并进一步讨论TM在评估该人群治疗效果中的临床意义。

## 1 材料和方法

### 1.1 研究对象

#### 1.1.1 LM组

回顾性分析2016年12月-2019年9月于南京鼓楼医院就诊的NSCLC伴LM患者，入组标准：①病理明确的NSCLC病史；②脑脊液细胞学找到恶性肿瘤细胞和/或新发的临床系统症状/体征合并典型的MRI影像学表现；③头颅MRI未见直径超过1 cm脑转移瘤病灶；④半年内无颅脑外伤、脑炎及脑部放疗史。其中32例诊断为LM，最终入组可获得同期CSF及血清TM的患者19例。所有患者均有靶点突变，前期接受过相关治疗（包括手术、靶向药物、多线化疗、免疫治疗等）。其中11例患者LM后植入Ommaya囊，间断引流CSF和接受鞘内化疗（表 1）。

**1:** LM组和NMNDs组患者临床特征比较 Clinical characteristics of the LM patients and NMNDs patients

Category	LM (*n*=19)	NMNDs (*n*=27)	*P*
Gendar (Male/Female)	6/13	10/27	0.702
Age (range, yr)	51.3 (39-62)	49.2 (38-69)	0.530
History of smoking			0.806
Previous or current smoking	5	8	
smoking			
No smoking	14	19	
Mean KPS (range)*	67.8 (30-90)	70.3 (30-90)	0.055
< 70	9	9	
≥70	10	18	
Clinical symptoms			
Headache/Nausea/Vomiting	13	21	0.513
Focal limb weakness	11	15	0.875
Cranial nerve abnormalities	6	9	0.901
Back pain	2	0	0.165
Urine incontinence	3	0	0.064
Seizure	5	3	0.246
Intracranial hypertension (> 180 cmH_2_O)	15	22	0.831
MRI abnormalities	13	26	0.089
*post leptomeningeal metastasis (LM); NMNDs: nonmalignant neurological diseases; MRI: magnetic resonance imaging.			

#### 1.1.2 对照组

收集同期南京鼓楼医院就诊的2, 258例Ⅳ期NSCLC患者（NSCLC组）血清TM、27例中枢神经系统良性肿瘤（nonmalignant neurological diseases, NMNDs）患者（NMNDs组）的CSF和血清TM作为对照，上述患者原发病均经过病理确诊，其中原发性中枢神经系统淋巴瘤患者22例，脑膜瘤患者5例。

### 1.2 实验方法

#### 1.2.1 脑脊液常规、生化检查及细胞学检测

所有LM组、NMNDs组患者均经过腰椎穿刺取得CSF，行CSF常规、生化及细胞学检测。记录CSF白细胞计数、中性粒细胞计数、淋巴细胞计数、红细胞计数、葡萄糖、氯化物以及CSF总蛋白。腰椎穿刺和经Ommaya囊引流CSF是LM患者诊断和治疗中的一部分，其中植入Ommaya囊患者共11例，在接受鞘内化疗患者每次治疗前留取经Ommaya囊引流的CSF，即脑室内CSF，完善相关指标检测，诊断和评估治疗疗效。经Ommaya囊取CSF时，常规消毒后用头皮针插入Ommaya囊内，抽出并丢弃囊和管内3 mL左右CSF，然后再留取7 mL左右CSF行检查评估。

#### 1.2.2 肿瘤标志物检测

采用一次性含分离胶及真空促凝剂管采集患者外周静脉血2 mL，3, 000 *g*，离心10 min，收集上清。采用无菌采样管收集CSF 3 mL。血清及CSF中TM均按全自动电化学发光免疫分析仪标准程序检测，分别检测：CEA、CA125、CYFRA21-1、NSE水平。所有标本都在收集2 h内完成检测。阳性判断标准：血清TM检测结果超过参考范围为阳性，即CEA > 4.7 ng/mL，CA125 > 30 U/mL，CYFRA21-1 > 5.5 ng/mL，NSE > 14.6 ng/mL。CSF中TM目前尚无标准的参考范围，考虑到正常情况下CSF中几乎检测不到TM，因此，将CSF中TM浓度高于血清参考范围作为阳性界值。

### 1.3 统计学处理

应用SPSS 22.0软件对数据进行统计学分析，呈正态分布的计量资料采用（Mean±SD）表示，配对设计的两样本均数比较采用配对*t*检验；计数资料采用百分数表示，组间比较采用*χ*^2^检验。*Kappa*检验行一致性分析。*P* < 0.05认为差异显著，有统计学意义。

## 2 结果

### 2.1 LM组与NMNDs组临床资料比较

LM组共纳入19例，男性6例，女性13例，年龄39岁-62岁，平均（51.3±4.9）岁。NMNDs组共27例，男性10例，女性17例，年龄38岁-69岁，平均（49.2±3.1）岁。LM组与NMNDs组之间性别、年龄、吸烟史、卡氏功能评分（Karnofsky performance status, KPS）、临床症状、颅内压增高比例均无统计学意义（*P* > 0.05）。此外，LM组13例（68.4%）观察到MRI异常，NMNDs组26例（96.3%）观察到MRI异常，但差异无统计学意义（*P*=0.089），详见表 1。

### 2.2 NSCLC组、LM组及NMNDs组TM水平比较

LM组4项血清TM水平及TM检出阳性率与NSCLC组差异无统计学意义（*P* > 0.05，见表 2）。与NMNDs组比较，无论是血清还是CSF中4项TM水平，LM组均明显高于NMNDs组，差异均有统计学意义（*P* < 0.001）。对于NMNDs组，血清与CSF中TM水平比较，差异无统计学意义（*P* > 0.05，图 1）。对于LM组，CSF中CEA、CYFRA21-1及NSE水平均高于血清，差异有统计学意义（*P* < 0.05），而CA125水平，CSF与血清中水平不具有统计学差异（*P* > 0.05），见图 1。

**2:** NSCLC组、LM组、NMNDs组的血清、脑脊液TM浓度比较 The comparison of serum/CSF levels of TM in NSCLC group, LM group and NMNDs group

Group	*n*	CEA (ng/mL)			CA125 (U/mL)			CYFRA21-1 (ng/mL)			NSE (ng/mL)	
		Serum	CSF		Serum	CSF		Serum	CSF		Serum	CSF
NSCLC	2258	72.2±351.1	-		155.1±200.1	-		9.2±38.2	-		22.89±42.62	-
LM	19	93.8±122.9	207.1±259.1		152.2±198.2	201.1±182.1		10.6±23.9	30.8±57.9		13.1±3.6	17.1±10.5
NMNDs	27	2.1±1.3	3.2±2.1		10.3±7.4	9.8±7.9		1.7±0.8	1.2±1.1		4.8±4.3	5.65±4.6
NSCLC: non-small cell lung cancer; CSF: cerebrospinal fluid; TM: tumor markers; CEA: carbohydrate antigen; CA125: carbohydrate antigen-125; CYFRA21: cytokeratin 19 fragments; NSE: neurone specific enolase.												

**1:**
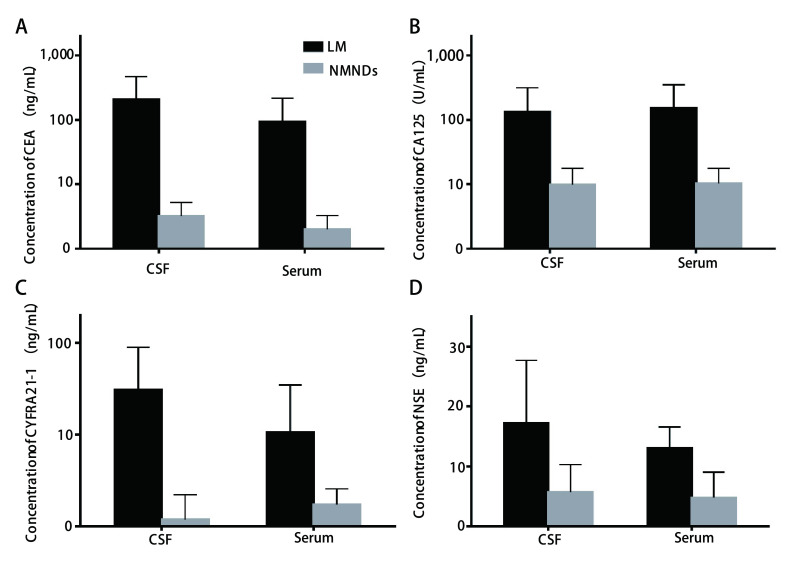
LM组与NMNDs组CSF及血清中四种TM的浓度比较（*P* < 0.05）。A: CEA；B: CA125；C: CYFRA21-1；D: NSE。 The comparison of the concentration of four TM between LM group and NMNDs group (*P* < 0.05). A: CEA; B: CA125; C: CYFRA21-1; D: NSE.

### 2.3 NMNDs组和LM组TM检出阳性率比较

对两组血清及CSF中TM阳性检出率进行分析比较：NMNDs组血清CEA、CA125、CYFRA21-1、NSE检出阳性率分别为7.4%、18.5%、14.8%、11.1%；CSF检出阳性率分别为11.1%、14.8%、7.4%、11.1%；NMNDs组血清与CSF中TM检出阳性率无统计学差异（*P* > 0.05）。LM组血清CEA、CA125、CYFRA21-1、NSE检出阳性率分别为63.2%、47.3%、47.4%、31.6%；CSF检出阳性率分别为68.4%、47.4%、63.2%、68.4%，LM组血清与CSF中TM检出阳性率差异无统计学意义（*P* > 0.05）。此外，与NMNDs组相比，LM组4项TM检出阳性率均明显高于NMNDs组，差异有统计学意义（*P* < 0.05）。

### 2.4 CSF中4种TM检测对LM辅助诊断价值

由表 3可见，LM组CSF中CYFRA21-1敏感性和阴性预测值最高，CEA特异性和阳性预测值最高。将CSF中4种TM进行了组合，联合检测时有任一项TM超过临界值既判定为阳性，则联合检测可提高辅助诊断LM的敏感性和阴性预测值。如果联合检测时所有TM超过临界值既判定为阳性，则可提高特异性和阳性预测值。在这些组合中，CEA或NSE任一项超过临界值则敏感性和阴性预测值100.0%，特异性74.1%。CYFRA21-1和NSE同时超过临界值时特异性和阳性预测值100.0%，敏感性78.9%。

**3:** CSF中TM检测对LM辅助诊断的相关指标比较（%） The comparison of four TM in CSF as an indicator for diagnosis of LM (%)

TM	Sensitivity	Specificity	Positive predictive value	Negative predictive value
CEA	85.0%	92.3%	89.5%	88.9%
CA125	77.8%	85.1%	73.7%	82.1%
CYFRA21-1	88.2%	86.2%	78.9%	92.6%
NSE	84.2%	88.9%	84.2%	88.9%
CEA+CA125^*^	94.7%	74.1%	72.0%	95.2%
CEA+CYF^*^	100.0%	70.4%	70.4%	100.0%
CEA+NSE^*^	100.0%	74.1%	73.1%	100.0%
CA125+CYF^*^	89.5%	70.4%	68.0%	90.5%
CA125+NSE^*^	89.5%	74.5%	70.8%	90.9%
CYF+NSE^*^	94.7%	74.1%	72.0%	95.2%
CEA+CA125+CYF^*^	100.0%	59.3%	63.3%	100.0%
CEA+CA125+NSE^*^	100.0%	63.0%	65.5%	100.0%
CEA+CYF+NSE^*^	100.0%	59.3%	63.3%	100.0%
CA125+CYF+NSE^*^	94.7%	59.3%	62.1%	94.1%
Four TM^*^	100.0%	48.1%	57.6%	100.0%
CEA+CA125^#^	63.2%	96.3%	92.3%	78.8%
CEA+CYF^#^	73.7%	100.0%	100.0%	84.4%
CEA+NSE^#^	68.4%	100.0%	100.0%	81.8%
CA125+CYF^#^	73.7%	100.0%	100.0%	84.4%
CA125+NSE^#^	68.4%	100.0%	100.0%	81.8%
CYF+NSE^#^	78.9%	100.0%	100.0%	87.1%
CEA+CA125+CYF^#^	63.2%	100.0%	100.0%	79.4%
CEA+CA125+NSE^#^	57.9%	100.0%	100.0%	77.1%
CEA+CYF+NSE^#^	63.2%	100.0%	100.0%	79.4%
CA125+CYF+NSE^#^	68.4%	100.0%	100.0%	81.8%
Four TM^#^	57.9%	100.0%	100.0%	77.1%
^*^The positive of combination was identified if concentration of any marker was positive; the negative of combination was identified if concentration of all markers was negative; ^#^The negative of combination was identified if concentration of any marker was negative; the positive of combination was identified if concentration of all markers was positive; CYF: CYFRA21-1.				

### 2.5 LM组不同亚组人群中TM检测结果的比较

本研究中CSF细胞学确诊人群即CSF(+)组共14例，MRI特异性改变人群即MRI(+)组共13例，其中MRI与CSF细胞学共同阳性人群即CSF(+)MRI(+)共8例。与MRI(+)组相比，CSF(+)组4种TM检出阳性率均高于MRI(+)组；其中，CSF(+)MRI(-)组人群CSF中TM检出阳性率高于CSF(+)MRI(+)人群以及CSF(-)MRI(+)，但差异不显著（*P* > 0.05）。CSF(+)组与MRI(+)血清TM检出阳性率差别不明显，同时CSF(+)MRI(-)、CSF(+)MRI(+)与CSF(-)MRI(+)人群血清TM检出阳性率无明显差别，均不具有统计学意义（*P* > 0.05），结果见图 2。

**2:**
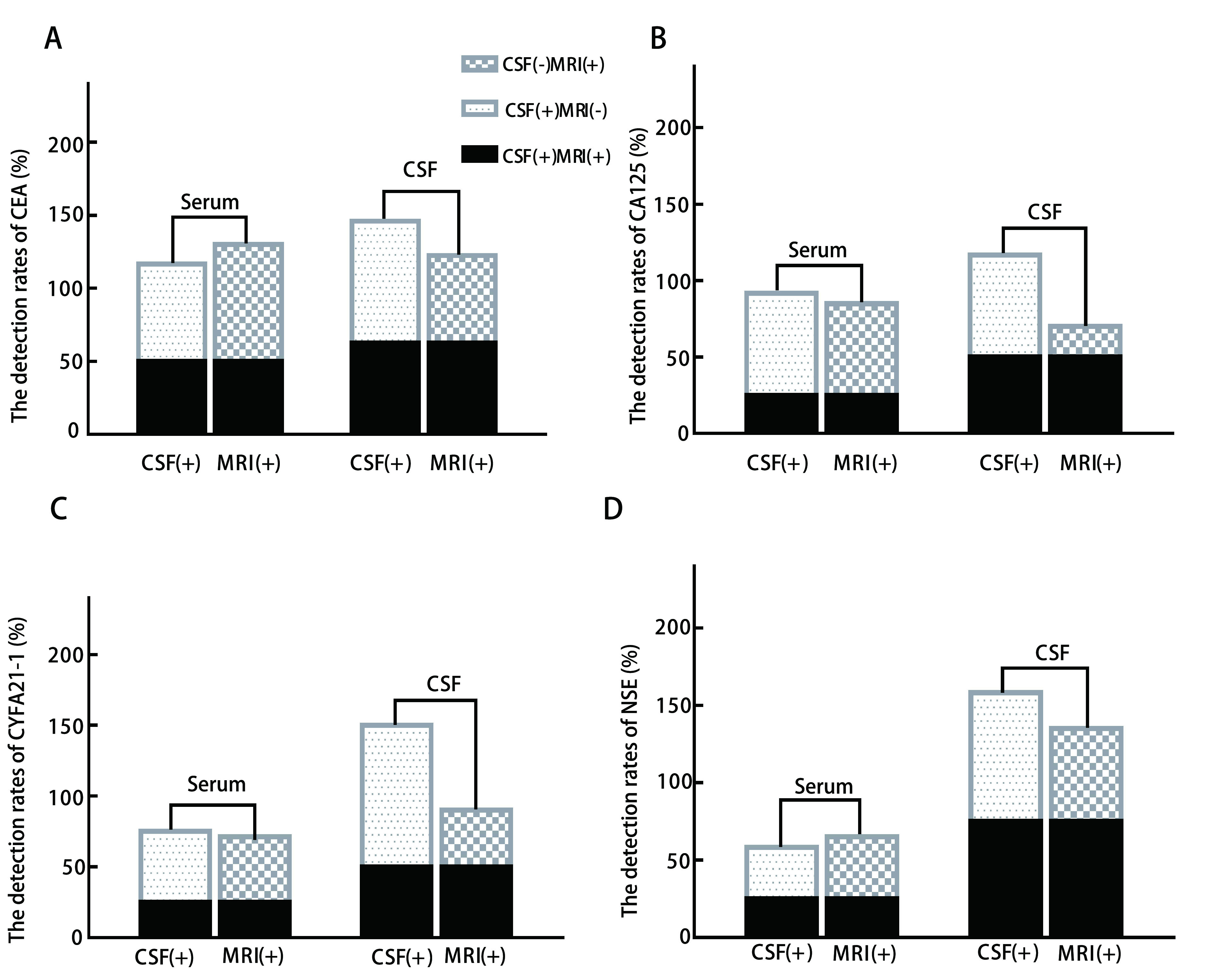
LM组不同人群中脑脊液和血清的TM检测阳性率的比较。A：脑脊液细胞学阳性人群与磁共振异常人群CEA的阳性率；B：脑脊液细胞学阳性人群与磁共振异常人群CA125的阳性率；C：脑脊液细胞学阳性人群与磁共振异常人群CYFRA21-1的阳性率；D：脑脊液细胞学阳性人群与磁共振异常人群NSE的阳性率。CSF(+)：脑脊液细胞学阳性人群。CSF(-)：脑脊液细胞学阴性人群。MRI(+)：磁共振异常人群。MRI(-)：磁共振无异常人群。 The detection rates of four TM in CSF and Serum in different populations of LM groups. A: The detection rates of CEA between CSF(+) and MRI(+); B: The detection rates of CA125 between CSF(+) and MRI(+); C: The detection rates of CYFRA21-1 between CSF(+) and MRI(+); D: The detection rates of NSE between CSF(+) and MRI(+). CSF(+): Patients with malignant tumor cells in CSF. CSF(-): Patients with no malignant tumor cells in CSF. MRI(+): Patients with abnormal MRI finds; MRI(-): Patients with normal MRI finds.

### 2.6 LM组血清与CSF中TM检测结果一致性分析

本研究采用线性加权kappa系数，分析血清与CSF中TM对19例NSCLC伴LM诊断的一致性，结果显示，血清与CSF中TM的检测情况对NSCLC伴LM诊断结果不一致，其中CEA、CA125、CYFRA21-1、NSE的加权kappa系数分别为0.038、0.578、0.066、0.163，不具有相关性（*P* > 0.05）（表 4）。

**4:** 四种TM的Kappa一致性分析 Kappa consistency analysis of four TM

Detection of TM	PCR (%)	NCR (%)	Kappa	P	95%CI
CEA	66.7	28.6	0.038	0.865	-0.403-0.479
CA125	77.8	80.0	0.578	0.012	0.210-0.945
CYFRA21-1	66.7	40.0	0.066	0.746	-0.360-0.491
NSE	83.3	38.5	0.163	0.342	-0.153-0.485
PCR: Positive consistency rate; NCR: Negative consistency rate.					

### 2.7 腰椎穿刺和脑室中CSF检测对比

经腰椎穿刺获取的CSF中，白细胞计数、淋巴细胞计数、葡萄糖、蛋白定量、氯化物、CEA、CA125、CYFRA21-1、NSE值稍高于脑室中CSF相关数值，但无统计学差异（*P* > 0.05），中性粒细胞计数两组无差异（表 5，*P* > 0.05）。

**5:** 经腰椎穿刺和脑室引流脑脊液对比 Comparison of assay results in CSF obtained from lumbar puncture and ventricular

	Lumber CSF (*n*=19)			Ventricular CSF (*n*=11)		
	Mean	Median (SD)		Mean	Median (SD)
WBC (×10^6^/L)	21.5	20.0 (12.5)		19.5	18.0 (13.7)
Lymphocyte (×10^6^/L)	16.1	13.0 (10.2)		13.2	10.9 (10.6)
Neutrophil (×10^6^/L)	5.53	3.0 (5.3)		6.3	3.0 (6.5)
Glucose (mmol/L)	1.9	1.9 (1.1)		1.8	1.2 (1.3)
Protein (g/L)	1.62	1.1 (1.3)		1.7	0.9 (1.6)
Chloride (mmol/L)	118.4	118.9 (6.0)		117.6	117.2 (5.5)
CEA (ng/mL)	175.4	69.1 (248.1)		136.5	52.2 (237.1)
CA125 (U/mL)	116.5	14.7 (171.3)		104.5	12.7 (165.1)
CYFCA21-1 (ng/mL)	26.8	4.6 (53.8)		29.4	4.2 (57.9)
NSE (ng/mL)	18.1	15.9 (12.3)		18.9	15.1 (13.9)
WBC: white blood cells.					

### 2.8 动态监测CSF中TM预测意义

动态监测治疗过程中CSF和血清TM变化，代表性结果如图 3所示。2例患者分别进行了8次和7次经Ommaya囊鞘内注射化疗。与血清TM相比，患者1的CSF中CEA和CA-125水平显著升高，在鞘内化疗后降至正常值，CSF细胞学提示恶性细胞完全被清除，随访中患者血清CEA和CA125不断增加，预示着颅外病灶的复发，而CSF中TM保持正常，与LM症状未复发一致（图 3A和图 3B）。患者2在诊断LM时CSF中CEA和CYFRA21-1升高，而相应的血清水平持续正常，并且CSF中TM水平持续增加，直到LM进展死亡为止（图 3C和图 3D）。该患者的CSF细胞学始终可查见恶性细胞。

**3:**
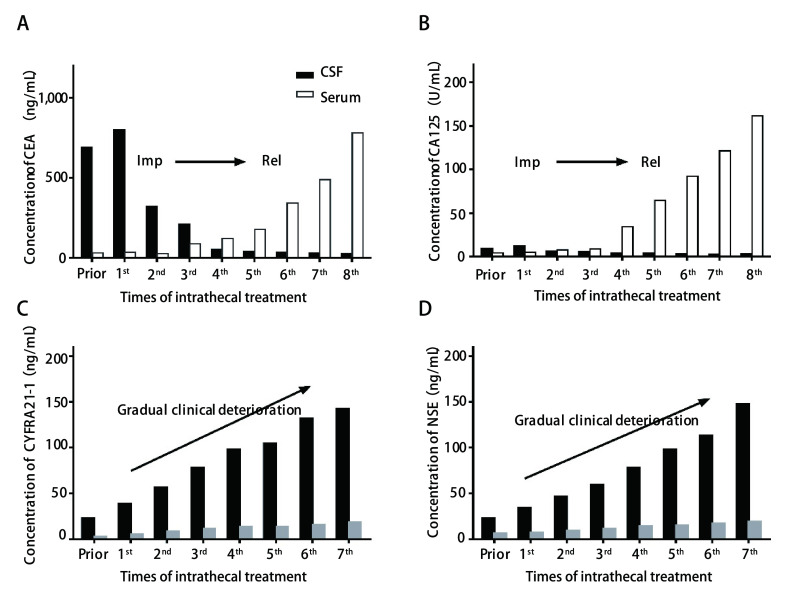
2例患者经Ommaya囊鞘内化疗前取脑脊液/血清监测肿瘤指标的动态变化。病例1（A和B）；病例2（C和D）。A：病例1的CSF中CEA水平高于血清，随着鞘内治疗有效CSF中CEA下降，最终血清CEA升高提示全身性（非中枢神经系统）复发；B：病例1的CSF和血清CA125差异不大，随着疾病进展CSF中CA125未见升高，血清CA125明显升高提示颅外进展；C：病例2诊断LM时CSF中CEA升高，而血清CEA基本正常，随着LM进展，CSF和血清中CEA水平明显增加，直到死亡之前CSF水平总是高于血清；D：随着LM进展，病例2血清和CSF中CYFRA21-1水平逐渐增加，直到死亡之前CSF水平总是高于血清。Imp：改善；Rel：复发。 CSF and serum TM levels were determined prior to each intrathecal chemotherapy via Ommaya reservoirs in 2 LM patients. Patient 1 (A-B) and Patient 2 (C-D). A: The CSF CEA levels were higher than the serum in patient 1, but with successful intrathecal chemotherapy, CEA levels decreased in CSF, while they increased in serum, heralding extrathecal lesions relapse; B: The CSF and serum levels of CA125 were not significantly different in patient 1. With the progression of the disease, CSF CA125 levels did not increase, while serum CA125 levels significantly increased, suggesting extracranial progression; C: The CSF CEA levels were increased at diagnosis of LM, while serum CEA levels were normal. Then both CSF and serum CEA levels increased over time due to the progression of LM, with CSF CEA levels persistently being higher than in serum until death; D: With the progession of LM, both the serum and CSF levels of CYFRA 21-1 gradually increased, meanwhile the levels of CYFRA21-1 in CSF were continuously higher than in serum until death. Imp: Improvement; Rel: Relapse.

## 3 讨论

LM是NSCLC最严重的并发症之一，致残率和致死率高^[7]^。其中50%以上LM患者最终死于LM进展，预后极差^[8]^。LM起病隐袭，临床症状缺乏特异性^[9]^，常常发现不及时，导致患者错过了最佳治疗时机。因此，对于LM的早期诊断、及时治疗，能够改善患者生活质量，延长肺癌患者生存时间。

目前，关于LM诊断的金标准仍然是CSF细胞学检出癌细胞，特异性高达100%，但敏感性仅50%^[1]^，反复腰椎穿刺可进一步提高敏感性^[10, 11]^，但患者常难以配合。虽然MRI异常有助于LM的诊断，但报道显示，约50%患者的MRI增强无法发现强化灶，或因MRI结果模棱两可，无法作为诊断依据^[12]^。本研究中，19例LM患者中有13例（68.4%）MRI见脑膜强化灶，14例（73.7%）CSF发现癌细胞，敏感性高于MRI检查。由此可见，临床上肺癌伴LM漏诊现象时有发生，需要引起临床重视，亟待提高NSCLC患者LM的诊断水平。肺癌4项CEA、NSE、CYFRA21-1和NSE是临床上常用于辅助诊断肺癌的指标^[13]^，也可以在CSF中进一步分析追踪，但其对肺癌伴LM的诊断价值如何有待进一步研究，因此，本研究探讨了血清及CSF中CEA、NSE、CYFRA21-1和NSE对肺癌伴LM的辅助诊断价值。

文献^[14]^报道，CSF中TM可用于LM的早期辅助诊断。CSF中TM的异常主要是因为^[15]^：①血脑屏障的破坏，血清中TM扩散至CSF中；②颅内恶性细胞产生释放入CSF中。本研究中，19例LM患者血清中TM水平及检出阳性率与肺腺癌Ⅳ期患者TM一致，而无论血清还是CSF中TM检测水平和检出阳性率均明显高于NMNDs组，且有显著差异性。由此可见，血清和CSF中TM可作为肺癌伴LM的辅助诊断及鉴别诊断的重要指标。血清和CSF中TM浓度同时升高很常见，但也有文献^[16]^报道，血清CEA升高的LM患者，常常并不同时出现CSF中CEA升高。本研究中，LM组CSF中TM浓度高于血清，且LM患者血清TM与CSF中TM检出情况不一致，提示CSF中TM的升高与鞘内合成相关，也较血清浓度更为显著，对于肺癌伴LM早期诊断具有一定的提示意义，对临床上疑似LM的所有患者均应进行CSF的检测，而不仅仅依赖于血清TM的情况。

TM是机体对癌细胞反应过程中合成和释放的一类物质。据报道，CSF中TM水平的高低与CSF中癌细胞数量呈正比^[17]^。在我们的研究中，与经CSF细胞学确诊人群相比，经MRI确诊人群CSF中TM检出阳性率更低，分析可能与细胞学阳性人群CSF中癌细胞数量更多相关。因此，临床实践中，针对MRI阳性但CSF细胞学阴性人群，可尝试其他癌症筛查手段或组合多种TM进一步辅助诊断。当然，CSF中不同TM诊断的敏感性不同^[14, 18, 19]^，本研究CSF中CYFRA21-1敏感性最高，达到88.2%，CEA特异性最高，达到92.3%，尤其是TM的联合检测能进一步提高LM诊断的敏感性和特异性，改善由于当前诊断方法如CSF细胞学或MRI手段不灵敏引起的治疗延迟。值得注意的是，临床上CSF中TM的升高常常早于MRI异常^[20]^，故对NSCLC患者，如出现可疑的LM症状者，任一项TM的异常都具有重要的提示作用，利用CSF中TM的指标，有助于诊断和治疗LM，对该亚群患者预后的改善具有非常重要的临床意义。总之，与CSF细胞学和MRI相比，寻找具有高敏感性CSF中TM对LM诊断可能更加有效。

鞘内化疗是LM有效治疗手段之一，经Ommaya囊脑室内注射和腰椎穿刺鞘内注射是LM鞘内化疗两种给药模式^[21, 22]^，其中经Ommaya囊脑室内注射具有操作便捷、可重复引流、反复给药、药物浓度更高更均匀等优点，专家更推荐经Ommaya囊脑室内化疗^[23]^。目前，关于不同穿刺部位的CSF中TM用于评估LM的诊断和治疗价值未见临床报道，本研究发现，Ommaya囊的LM患者共11例，其CSF常规、生化、TM检测浓度与经腰椎穿刺检测水平无明显差异，结果证明经Ommaya囊脑室内CSF与腰椎穿刺CSF同样具有诊断价值，可作为肺癌LM诊断和鉴别诊断的重要指标，且在治疗过程中可动态监测CSF中相关指标，用于评估治疗疗效。

纵向的评估可以更有效检测疾病活动和治疗反应，对于LM患者的疗效评价至关重要。LM患者疗效评价主要依赖于RANO评分、CSF中细胞学以及影像学MRI。但存在以下局限：①RANO主要根据患者神经系统功能的缺失进行评分，研究报道^[24]^显示，LM患者治疗主要表现为颅高压得到改善，而神经系统功能缺失难以逆转；且RANO主观性强，易受干扰^[25]^。②有研究者利用液体活检检测CSF中恶性细胞是否清除^[26]^，但没有证据表明CSF中细胞学转阴与LM患者临床获益相关，同时该检查具有创伤性，敏感性低^[27]^，阴性结果仅代表检测的CSF的结果，并不能证明蛛网膜下腔或脑膜中病灶的缩小，故用CSF细胞学检测作为评估LM治疗有效并不可靠。③LM患者中20%-30%的MRI是正常的，其次LM的病灶在MRI上主要表现为结节、强化增厚，病灶无法测量^[28]^；同时LM患者的治疗往往清除的是漂浮在CSF中的恶性细胞，对于LM病灶往往无法有效清除^[29]^，故影像学的疗效评价仍存在挑战。

临床工作中常常将血清TM纵向变化用于复发判定、疗效评估，也有小型临床研究连续监测CSF中TM水平评估治疗反应^[30]^。本研究中有2例LM患者周期性经Ommaya囊脑室内化疗中，监测CSF和血清中TM的纵向变化，分别评估颅内和颅外肿瘤的疗效反应，可操作性强，且具有较好的临床指导意义。因此，监测血清及CSF中TM有助于选择可能从进一步的治疗中获益的人群。但本研究样本量偏少，且均为腺癌，因此，CSF中TM是否对不同病理类型的肺癌同样具有指导作用，不同的TM对LM中不同人群是否检测意义不同，以及血清和CSF中TM是否能够分别代表颅外和颅内纵向预测评估的指标，仍有待于后期更大规模样本的研究。

综上所述，血清及CSF中TM的检测可提高LM诊断的敏感性，且不同途径CSF中的TM均具有诊断价值和鉴别能力。TM不仅仅是NSCLC患者早期LM辅助诊断的重要指标，动态观察CSF中TM的演变规律也有助于临床判断病情进展、治疗反应以及评估预后的重要参考因素，当然，相关研究结论还需要更多临床数据进一步评估验证。
